# The role of PET/CT in disease activity assessment in patients with large vessel vasculitis

**DOI:** 10.1093/rheumatology/keac125

**Published:** 2022-03-08

**Authors:** Elena Galli, Francesco Muratore, Pamela Mancuso, Luigi Boiardi, Chiara Marvisi, Giulia Besutti, Lucia Spaggiari, Massimiliano Casali, Annibale Versari, Paolo Giorgi Rossi, Carlo Salvarani

**Affiliations:** Rheumatology Unit, Azienda Unità Sanitaria Locale-IRCCS di Reggio Emilia, Reggio Emilia; Rheumatology Unit, University of Modena and Reggio Emilia, Modena; Rheumatology Unit, Azienda Unità Sanitaria Locale-IRCCS di Reggio Emilia, Reggio Emilia; Epidemiology Unit; Rheumatology Unit, Azienda Unità Sanitaria Locale-IRCCS di Reggio Emilia, Reggio Emilia; Rheumatology Unit, Azienda Unità Sanitaria Locale-IRCCS di Reggio Emilia, Reggio Emilia; Rheumatology Unit, University of Modena and Reggio Emilia, Modena; Radiology Unit; Radiology Unit; Nuclear Medicine Unit, Azienda Unità Sanitaria Locale-IRCCS di Reggio Emilia, Reggio Emilia, Italy; Nuclear Medicine Unit, Azienda Unità Sanitaria Locale-IRCCS di Reggio Emilia, Reggio Emilia, Italy; Epidemiology Unit; Rheumatology Unit, Azienda Unità Sanitaria Locale-IRCCS di Reggio Emilia, Reggio Emilia; Rheumatology Unit, University of Modena and Reggio Emilia, Modena

**Keywords:** PET/CT, PETVAS, Takayasu arteritis, GCA, large vessel vasculitis

## Abstract

**Objectives:**

To evaluate the accuracy of PET/CT and of PET vascular activity score (PETVAS) in assessing disease activity and the ability of PETVAS in predicting relapses in a large single-centre cohort of patients with large vessel vasculitis (LVV).

**Methods:**

We conducted a retrospective cohort study of prospectively collected data of consecutive patients diagnosed with LVV who underwent at least one PET/CT scan between 2007 and 2020. The nuclear medicine physician’s interpretation of each PET/CT scan (active/inactive vasculitis) was compared with disease activity clinical judgement (active disease/remission). For each PET/CT scan, the PETVAS score was calculated and its accuracy in assessing disease activity was evaluated. The ability of PETVAS in predicting subsequent relapses was evaluated.

**Results:**

A total of 100 consecutive LVV patients (51 large vessel GCA, 49 Takayasu arteritis) underwent a total of 476 PET/CT scans over a mean follow-up period of 97.5 months. Physician-determined PET/CT grading was able to distinguish between clinically active and inactive LVV with a sensitivity of 60% (95% CI 50.9, 68.7) and specificity of 80.1% (95% CI 75.5, 84.1); the area under the curve (AUC )was 0.70 (95% CI 0.65, 0.75). PETVAS was associated with disease activity, with an age and sex–adjusted odds ratio for active disease of 1.15 (95% CI 1.11, 1.19). A PETVAS ≥10 provided 60.8% sensitivity and 80.6% specificity in differentiating between clinically active and inactive LVV; the AUC was 0.73 (95% CI 0.68, 0.79). PETVAS was not associated with subsequent relapses, with an age and sex–adjusted hazard ratio of 1.04 (95% CI 0.97, 1.11).

**Conclusions:**

The visual PET/CT grading scale and PETVAS had moderate accuracy to distinguish active LVV from remission. PETVAS did not predict disease relapses.

Rheumatology key messages

- The visual PET/CT grading scale and PETVAS have moderate accuracy to distinguish active LVV from remission.
- PET/CT shows better performance to distinguish active disease from remission in TAK than in LV-GCA.
- PETVAS does not predict disease relapses.

## Introduction

GCA and Takayasu arteritis (TAK) are the two main forms of large vessel vasculitis (LVV) that share many clinical, pathological and radiographic features [1, 2]. Despite many attempts to adopt standardized approaches for assessing disease activity in LVV, no single measure or set of measures have been accepted as valid and useful in clinical practice [3].

^18^F-fluorodeoxyglucose (^18^F-FDG) PET combined with CT, hereafter termed PET/CT, is one of the most sensitive and specific diagnostic tools in LVV [4]. PET/CT is also useful in identifying the presence of occult LVV, particularly in patients with PMR who have persistence of symptoms despite treatment with glucocorticoids (GCs) or in those with atypical PMR [5, 6]. The utility of PET/CT in diagnosing LVV has been confirmed by several studies, however, its utility to monitor disease activity or predict relapse remains unclear [7–11].

Many visual/qualitative and semiquantitative PET interpretation criteria have been proposed in LVV [11]. The visual grading scale (vascular to liver uptake) is reproducible and easy to use in clinical practice, however, the regional uptake information from specific single lesions provided by these criteria may not be sufficient to define the whole-body burden of inflammation and for assessing disease activity and treatment response [12]. The PET vascular activity score (PETVAS) is a newly developed PET-based parameter created by integrating the visual 0–3 scores of nine main susceptible arteries, which can quantitatively better reflect the global inflammatory burden [13]. PETVAS has been proven to be useful to differentiate clinically active and inactive disease, predict disease relapses and monitor treatment response in LVV patients [12–15].

The aims of the present study were to evaluate the accuracy of PET/CT and PETVAS in disease activity assessment in a large single-centre cohort of patients with LVV and to evaluate the ability of PETVAS in predicting relapses in the same cohort.

## Patients and methods

### Study design and population

This is a retrospective cohort study based on prospectively collected data of consecutive patients diagnosed with LVV who were referred to the Rheumatology Unit of the Santa Maria Nuova Hospital of Reggio Emilia and underwent at least one PET/CT scan at our institution between January 2007 and December 2020. In all patients, the diagnosis of LVV was confirmed by imaging and all patients satisfied the modified inclusion criteria of the Giant Cell Arteritis Actemra (GiACTA) trial for GCA or the 1990 ACR classification criteria for TAK [16, 17]. Patients were enrolled at various stages during the disease, but PET/CT scans were preferentially performed during periods of clinically active disease or when taking <15 mg/day prednisone during clinical remission. For the first objective, all patients were included. For the second objective, only patients who underwent a PET/CT scan during a period of clinical remission and had at least 6 months of clinical follow-up were included.

### Disease assessment and clinical management

Since 2007, patients with LVV have received a standardized yearly evaluation of disease activity and extension by means of colour duplex ultrasonography (CDUS), CT angiography (CTA) or magnetic resonance angiography (MRA) and PET/CT [18]. The standard follow-up procedure includes a clinical rheumatological visit and acute phase reactant determination every 3 months. Additional CDUS, CTA or MRA and PET/CT could be requested in case of flare or evolving vascular damage. This clinical protocol includes determination of acute phase reactants and complete physical examination performed by a trained rheumatologist 24 or 48 h prior to imaging assessment.

### Outcome definition and medical records review

The available medical records of the study participants were retrospectively reviewed from the date of LVV diagnosis to the end of the study period (31 December 2020), last follow-up or death. Information about demographics, clinical manifestations, imaging and laboratory findings and GC doses were collected at diagnosis and at each follow-up visit. Disease activity was evaluated using the Kerr/National Institutes of Health index, which assesses four items: constitutional manifestations, elevated ESR and/or CRP, clinical manifestations of vascular ischaemia or inflammation and angiographic features indicative of vasculitis. Disease was defined as active in the presence of at least two new or worsened items in the previous 3 months [19]. For the purposes of this study we used CTA or MRA to determine vessel lumen changes instead of conventional digital subtraction angiography. In patients with LV-GCA, disease was considered active also in the presence of unequivocal cranial and/or visual symptoms and/or PMR associated with elevated ESR or CRP. Remission was defined as the absence of any clinical symptoms directly attributable to vasculitis. Isolated fatigue was not considered a vasculitic symptom. A disease relapse was defined by all of the following: reappearance of signs/symptoms attributed to vasculitis, resolution of signs/symptoms after increasing or restarting GC, ESR ≥40 mm/h or CRP ≥0.5 mg/dl and exclusion of other causes. We also considered relapses as the appearance of new lumen changes in a previously unaffected territory or new changes in an area already involved by vasculitis at follow-up CTA, MRA or CDS or new/increased FDG uptake at follow-up PET/CT associated with a variation in the GC dose and/or immunosuppressive treatment, even in the absence of any other symptom or sign or increase in inflammatory markers. Isolated elevation of acute phase reactant levels was not considered active disease or relapse. Time to relapse was calculated from the date of the first PET/CT scan performed in clinical remission to the date of the first subsequent relapse.

### PET/CT imaging protocol and review

All PET-CT examinations were acquired by using a hybrid PET/CT machine (Discovery, GE Healthcare, Chicago, IL, USA) with 3.30 min emission scan/bed and CT-attenuation correction. Patients were required to fast for at least 4 h before i.v. injection of 37 MBq of ^18^F-FDG per 13 kg of patient weight. Blood glucose levels before tracer injection were <200 mg/ml in all cases. All patients were in the supine position with their arms alongside the body. A low-dose, non-contrast-enhanced CT scan was carried out for PET co-registration. A whole-body emission scan was performed 60 min after ^18^F-FDG injection from the base of skull to the proximal femora [11].

All identified PET-CT scans were reviewed by a single nuclear medicine physician with expertise in LVV (MC) who was blinded to clinical and morphological imaging data and previous PET/CT reports. The nuclear medicine physician determined whether the findings were consistent with active or inactive vasculitis using the visual 0–3 vascular to liver FDG uptake grading scale: 0 = no uptake (≤ mediastinum); 1 = low-grade uptake (< liver); 2 = intermediate-grade uptake (= liver), 3 = high-grade uptake (> liver). Scans showing grade 3 and 2 FDG uptake in any large artery other than femoral arteries were classified as ‘active’; scans showing grade 1 and 0 FDG uptake were classified as ‘inactive’ (physician-determined PET/CT grading) [11]. Additionally, the scans were graded by visual assessment of FDG uptake in four segments of the aorta (ascending, arch, descending thoracic and abdominal) and in five branch arteries (carotids, brachiocephalic trunk, subclavian arteries). The degree of FDG uptake in these arteries was visually assessed relative to the FDG uptake of the liver and a PETVAS of 0–27 points was calculated by adding the qualitative scores of the nine arterial territories [13]. In a recent study involving two centres, in which two nuclear medicine specialists (including the one involved in the present study, MC) reviewed the PET/CT scans of patients with LVV by using the 0–3 visual grading scale and the PETVAS, interreader reliability was >95% [15].

### Statistical analysis

Continuous variables are reported as mean and s.d. and categorical variables as proportions. The intergroup difference was compared by using the *t*-test and χ^2^ test.

The association between the PETVAS and disease activity was evaluated by means of a Cuzick non-parametric test for trend across order groups (NPtrend). Analyses were conducted using each PET examination as a statistical unit, but taking into account the intra-individual correlation to obtain robust variance estimates with the SVY command in STATA/IC version 13 (StataCorp, College Station, TX, USA) in logistic regression.

The Cox proportional hazards model was used to assess the relation between the PETVAS and subsequent relapse; the PETVAS was treated as a time-dependent variable changing every time a new PET assessment was available. We reported age and sex–adjusted hazard ratios (HRs) and 95% CIs; in order to account for right censoring, 9 months of survival were added after the last negative clinical follow-up assessment.

The predictive ability of the PETVAS was assessed by means of receiver operating characteristics (ROC) curves reporting the areas under the curve (AUCs) for disease activity and subsequent relapse.

The study was approved by the Ethics Committee of the Area Vasta Emilia Nord (protocol 2020/0089366). Given the retrospective nature of the study, the Ethics Committee authorized the use of a patient’s data without his/her informed consent if all reasonable efforts had been made to contact that patient.

## Results

### Characteristics of the study population

**Fig. 1:**
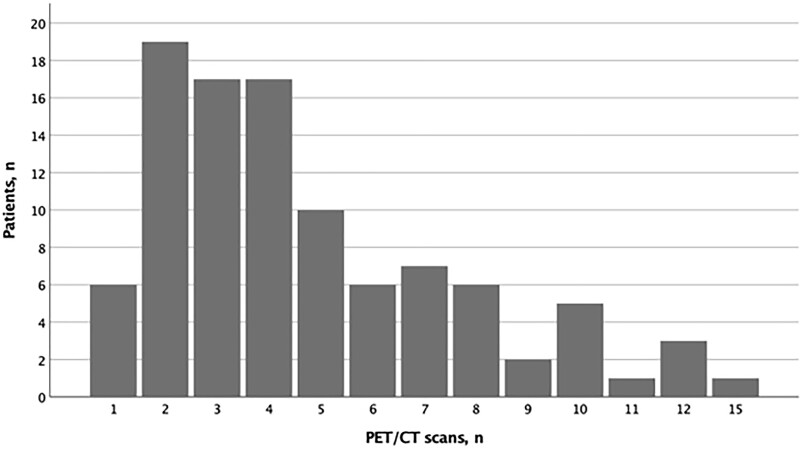
Distribution of PET/CT scans in the study cohort

In the study period, 100 consecutive LVV patients (51 LV-GCA, 49 TAK) underwent a total of 476 PET/CT scans over a mean follow-up period of 97.5 months (s.d. 60.2). Each patient underwent a mean of 4.8 (s.d. 3.0) PET scans. The distribution of the PET/CT scans in the study cohort is shown in Fig. 1.

Demographic and baseline characteristics of the study population are presented in Table 1. The mean age at diagnosis was 48.1 years and 79% of patients were female. The first PET/CT scan was performed at disease diagnosis in 46% of patients (newly diagnosed LVV). The mean disease duration at PET/CT scans was 60 months and the mean PETVAS was 6.

**Table 1:** Demographic and baseline characteristics of the study population

Characteristics	LV-GCA	TAK	Total
Patients			
Patients, *n* (%)	51 (51)	49 (49)	100 (100)
Age at diagnosis, mean (s.d.), years	64.7 (9.3)	30.9 (10.7)	48.1 (19.6)
Female, *n* (%)	36 (70.6)	43 (87.8)	79 (79)
Newly diagnosed at first PET/CT, *n* (%)	32 (62.7)	14 (28.6)	46 (46)
Time between symptoms onset and diagnosis, mean (s.d.), months	10.5 (26.3)	24.4 (48.4)	17.3 (39.2)
Disease duration at first PET/CT scan, mean (s.d.), months	9.1 (16.8)	41.9 (57.2)	25.2 (44.7)
Follow-up, mean (s.d.), months	83.9 (47.9)	111.6 (68.5)	97.5 (60.2)
PET/CT scans			
PET/CT scans, *n* (%)	249 (52.3)	227 (47.7)	476 (100)
Disease duration at PET/CT scans, mean (s.d.), months	52.3 (47.1)	68.5 (61.6)	60.1 (54.9)
Clinically active disease, *n* (%)	71 (28.5)	54 (23.8)	125 (26.3)
Active PET/CT scans (physician-determined), *n* (%)	67 (26.9)	78 (34.4)	145 (30.5)
PETVAS, mean (s.d.)	5.9 (7.5)	6.1 (5.9)	6.0 (6.8)

A total of 26% of PET/CT scans were performed in patients with clinically active LVV (Table S1). The mean PETVAS was 10.4 (s.d. 9.2) in clinically active LV-GCA and 10.5 (s.d. 5.7) in clinically active TAK and 4.1 (s.d. 5.7) in clinically inactive LV-GCA and 4.6 (s.d. 5.1) in clinically inactive TAK (*P* < 0.05 for both). There was no difference in the mean PETVAS between clinically active LV-GCA *vs* TAK and clinically inactive LV-GCA *vs* TAK.

### Accuracy of PET/CT

Of 476 PET/CT scans, 145 (30.5%) were interpreted as consistent with active vasculitis by a nuclear medicine physician (Table 1). The proportions of patients who had a PET/CT scan interpreted by a nuclear medicine physician as active vasculitis were as follows: 75/125 with clinically active LVV and 70/351 with LVV in remission. Physician-determined PET/CT grading was able to distinguish between patients with clinically active LVV and patients with LVV in clinical remission with a sensitivity of 60% (95% CI 50.9, 68.7) and a specificity of 80.1% (95% CI 75.5, 84.1). The positive likelihood ratio was 3.01 (95% CI 2.33, 3.88) and the negative likelihood ratio was 0.50 (95% CI 0.40, 0.62).

The AUC of physician-determined PET/CT grading in differentiating between clinically active and inactive LVV was 0.70 (95% CI 0.65, 0.75). The following sensitivity, specificity, positive likelihood ratio and negative likelihood ratio were found in the LVV subgroups: 50.7% (95% CI 38.6, 62.8), 82.6% (95% CI 76.2, 87.8), 2.91 (95% CI 1.96, 4.32) and 0.60 (95% CI 0.47, 0.76) for LV-GCA and 72.2% (95% CI 58.4, 83.5), 77.5% (95% CI 70.5, 83.5), 3.20 (95% CI 2.32, 4.42) and 0.36 (95% CI 0.23, 0.56) for TAK, respectively. The AUC of physician-determined PET/CT grading in differentiating between clinically active and inactive disease was 0.67 (95% CI 0.60, 0.73) for LV-GCA and 0.75 (95% CI 0.68, 0.82) for TAK.

Compared with patients with LVV in clinical remission, those with clinically active disease had significantly higher PETVAS, ESR levels and CRP levels and more frequently were on prednisone therapy, taking higher prednisone doses and had shorter disease duration at the time of PET/CT scans (Table 2).

**Table 2:** Comparison between clinically active and inactive LVV patients

Characteristics	Active LVV (*n* = 125)	LVV in remission (*n* = 351)	*P*-value
Age at PET/CT, mean (s.d.), years	49.9 (19.1)	51.0 (16.8)	0.534
PETVAS, mean (s.d.)	10.5 (7.9)	4.4 (5.5)	<0.0001
ESR, mean (s.d.), mm/1 h^a^	54.6 (34.5)	20.2 (15.1)	<0.0001
CRP, mean (s.d.), mg/dl^b^	5.4 (10.4)	0.8 (1.4)	<0.0001
Presence of at least one symptom suggestive for active vasculitis, *n* (%)^c^	95 (76.0)^d^	0^e^	<0.0001
Patients on prednisone, *n* (%)	111 (88.8)	191 (54.4)	<0.0001
Prednisone dose, mean (s.d.), mg/day	31.6 (19.1)	11.2 (11.9)	<0.0001
Patients on prednisone >20 mg/day, *n* (%)	71 (56.8)	31 (8.8)	<0.0001
Disease duration at PET/CT scans, mean (s.d.), months	37.4 (49.9)	68.1 (37.4)	<0.0001

a Missing = 16.

b Missing = 11.

c Missing = 8.

d 30 patients without any symptoms suggestive for active vasculitis were classified as active according to the Kerr/NIH criteria (elevated ESR and/or CRP and angiographic features indicative of vasculitis).

e 13 patients (3.7%) in LVV remission had isolated fatigue associated with elevated ESR or CRP.

Absent vascular FDG uptake (PETVAS 0) was observed in 207 (58.3%) PET/CT scans performed during disease remission (13 performed on prednisone >20 mg/day) and in 34 (28.1%) PET/CT scans performed during clinically active disease (10 performed on prednisone >20 mg/day).

The PETVAS was associated with disease activity: age and sex–adjusted OR for each unit increase of PETVAS of having active disease was 1.15 (95% CI 1.11, 1.19). The association was stronger in TAK patients than in LV-GCA patients [OR 1.23 (95% CI 1.14, 1.33) and 1.12 (95% CI 1.07, 1.17), respectively].

**Fig. 2:**
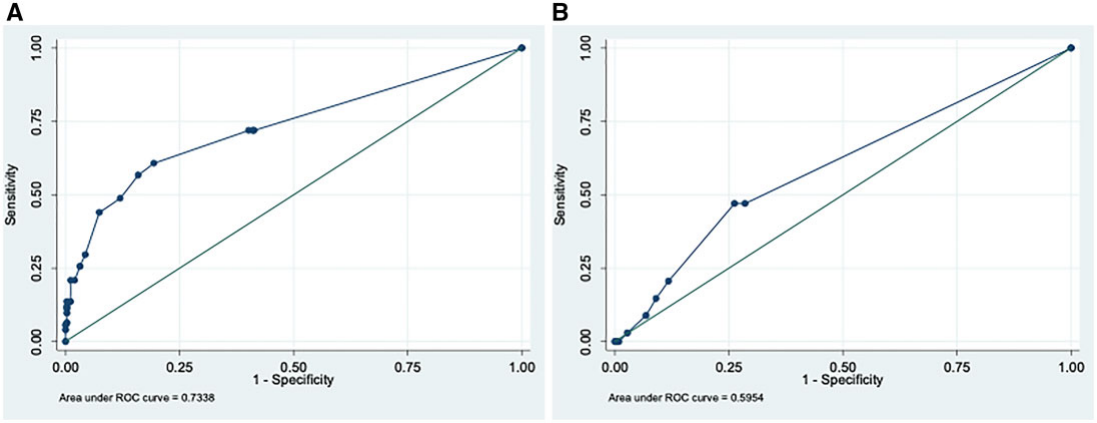
AUC of the PETVAS in distinguishing active LVV from remission and predicting subsequent relapses. (**A**) AUC of the PETVAS in differentiating between clinically active and inactive LVV. (**B**) AUC of the PETVAS in predicting subsequent relapses in LVV.

The AUC of the PETVAS in differentiating between clinically active and inactive LVV was 0.73 (95% CI 0.68, 0.79) (Fig. 2A). The AUC was higher in TAK than in LV-GCA patients [AUC 0.79 (95% CI 0.71, 0.87) and 0.70 (95% CI 0.62, 0.78), respectively]. A PETVAS ≥10 provided 60.8% sensitivity and 80.6% specificity in differentiating between clinically active and inactive LVV. A PETVAS ≥10 provided the following sensitivity and specificity in differentiating between clinically active and inactive LVV subgroups: 52% and 82% in LV-GCA and 73% and 78% in TAK, respectively.

### Value of PETVAS for predicting subsequent relapses in LVV

Eighty-one patients with LVV who underwent a PET/CT scan during a period of clinical remission were included for the analysis on relapses. A total of 34 clinical relapses were observed (19 relapses in TAK and 15 in LV-GCA); the mean time to relapse was 14.6 months (Supplementary Table S1, available at *Rheumatology* online). Between the first PET/CT performed in clinical remission and the first subsequent relapse, seven patients modified the ongoing treatment (five patients suspended GCs and two patients suspended methotrexate). The PETVAS was not associated with subsequent relapses [age and sex–adjusted HR 1.04 (95% CI 0.97, 1.11), *P* = 0.252]. The AUC of the PETVAS in predicting subsequent relapses was 0.60 (95% CI 0.50, 0.69) (Fig. 2B). A PETVAS ≥9 showed 47.1% sensitivity and 73.8% specificity in predicting subsequent relapses. Similar results were observed when the analyses were performed in LVV subgroups (Supplementary Materials, available at *Rheumatology* online).

## Discussion

We evaluated the role of PET/CT in assessing disease activity and in predicting subsequent relapses in a large single-centre cohort of LVV patients with long-term follow-up. The main findings were that the visual PET/CT grading scale and the PETVAS have moderate accuracy to distinguish active disease from remission and that the PETVAS does not predict subsequent disease relapses.

In the present study, the visual PET/CT grading scale was able to distinguish between clinically active LVV and LVV in clinical remission with a sensitivity of 60% and a specificity of 80%; the AUC was 0.70, indicating moderate accuracy. In a recent meta-analysis of four cross-sectional studies (111 LVV patients with 136 scans), PET/CT showed a pooled sensitivity of 77% (95% CI 57, 90) and a specificity of 71% (95% CI 47, 87) [9]. It is of interest that our monocentric study showed similar results despite the heterogeneity of the studies included in the meta-analysis. Another recent meta-analysis of nine studies (298 LVV patients with 439 scans) reported higher accuracy, with a pooled sensitivity estimate of PET/CT for the detection of active LVV of 88% (95% CI 79, 93) and the pooled specificity of 81% (95% CI 64, 91); the AUC was 0.91 (95% CI 0.89, 0.94) [20]. It is important to note that all nine studies included in this meta-analysis enrolled TAK patients, whereas only one also included GCA. Similarly, when we evaluated LVV subgroups, we found that the visual PET/CT grading scale had a better performance in distinguishing between clinically active and inactive disease in TAK (72% sensitivity, 78% specificity, AUC 0.75) than in LV-GCA (51% sensitivity, 83% specificity, AUC 0.67). Similar results were reported by a meta-analysis of seven studies that included 191 TAK patients, showing a pooled sensitivity of 87% (95% CI 78.0, 92.6) and pooled specificity of 73% (95% CI 62.5, 81.3) for the assessment of disease activity [10]. All these results are in line with previous studies and suggest a stronger correlation between FDG uptake and clinical disease activity in TAK compared with GCA.

The concept of the heterogeneity of GCA with different subsets characterized by different clinical manifestations is widely accepted [21–24]. At one end of the spectrum there are patients with temporal artery involvement with prevalent cranial manifestations, while at the other end there are patients with large vessel involvement with prevalent systemic manifestations and polymyalgic symptoms; patients with overlapping features stay in the middle [21, 24]. Most of the studies (including the present) that evaluated the performance of PET/CT in the assessment of disease activity included in the definition of active disease the entire spectrum of clinical manifestations of GCA (cranial, systemic, visual and polymyalgic symptoms) [9, 10, 20]. However, these manifestations are not always an expression of the large vessel inflammation evaluated by PET/CT (i.e. cranial symptoms are an expression of temporal artery inflammation, which is not visualized by PET/CT, and PMR is an expression of bursitis and tenosynovitis). We believe that this may represent one of the possible explanations for the lower sensitivity of PET/CT in differentiating active and inactive disease in GCA compared with TAK reported by most studies. The younger age and the lower prevalence of atherosclerosis in TAK could be other explanations.

PET/CT has been used in the follow-up of patients with LVV. In this regard, in a series of 21 patients who experienced clinical improvement following therapy, the target:background ratio (TBR; aortic wall uptake divided by blood pool uptake) also decreased [25]. However, persistence of FDG vascular uptake is frequently observed in patients with LVV, even in those treated with anti-IL-6 therapy [15]. In this regard, a recent study on 30 tocilizumab-treated patients with LVV who underwent PET/CT scans due to active disease before tocilizumab use showed that although most of them achieved clinical remission following therapy, less than one-third showed normalization of FDG vascular uptake [26].

In our cohort, 20% of PET-CT scans performed in patients with LVV in clinical remission were interpreted as active vasculitis by a nuclear medicine physician, a proportion lower than that reported by Grayson *et al.* [13] (58%). This may be caused by divergence of visual judgement standards and by the delayed uptake time of 2 h used by Grayson *et al.* compared with a 1 h uptake time used in the present study. Delay in the time interval from injection of FDG to image acquisition can increase the sensitivity for detecting FDG uptake in the arterial wall by allowing more time for distribution of FDG into tissue, with concomitant elimination from the blood pool [13, 27, 28]. However, persistent low-grade vascular FDG uptake was observed in 42% of our PET/CT scans performed during disease remission. It is unclear whether this persistent low-grade metabolic activity in the vascular wall detected in patients with LVV during clinical remission represents subclinical vasculitis, remodelling, atherosclerosis or a combination of these factors.

After the initiation of an adequate dose of GC treatment, vascular FDG uptake rapidly decreases, reducing the diagnostic accuracy of PET/CT [29]. In a recent prospective study, authors showed that the sensitivity of PET/CT in diagnosing LV-GCA is high within 3 days of high-dose GC treatment, while it significantly decreases after 10 days of treatment [30]. Therefore the accuracy of PET/CT and PETVAS in discriminating between active and inactive disease in longitudinal studies may be influenced by GC dosage. Our data have to be interpreted with caution, because we cannot exclude that GC treatment significantly influenced our results, particularly in the patients with active LVV who were on a high dose of prednisone when they underwent PET/CT.

Our study confirms that the PETVAS is a simple qualitative metric of arterial FDG uptake that quantitatively reflects the global inflammatory burden. The mean PETVAS found in our study is similar to that reported by Kang *et al.* [12] but lower than that reported by the original study from the National Institutes of Health (NIH) [13]. As suggested by Kang *et al.* this may be caused by divergence of visual judgement standards. Detailed standardization should be established, especially in iterative reconstruction methods, pseudo-colour selection, scale bar setting, display levels of liver reference points and determination of scoring, in order to increase interinstitution reproducibility [12]. Differently from Grayson *et al.* [13], we did not find a significant difference in the mean PETVAS between TAK and GCA patients.

The PETVAS showed moderate performance for the assessment of disease activity in LVV in both our study (AUC 0.73, with a cut point of ≥10 yielding a sensitivity of 61% and a specificity of 81%) and the original study by the NIH (AUC 0.72, with a cut point of ≥20 yielding a sensitivity of 68% and a specificity of 71%) [13]. When we evaluated LVV subgroups, we found better performance of the PETVAS for the assessment of disease activity in TAK (AUC 0.79, with a cut point of ≥10 yielding a sensitivity of 73% and a specificity of 78%) than in LV-GCA (0.70, with a cut point of ≥10 yielding a sensitivity of 52% and a specificity of 82%). Our data are in line with those by Kang *et al.* [12], who assessed the performance of the PETVAS in a cohort of 54 TAK patients who underwent a total of 64 PET/CT scans and reported an AUC of 0.86, with a cut point of ≥8.5 yielding a sensitivity of 76% and a specificity of 83% in assessing disease activity.

Finally, we did not find an association between the PETVAS and the subsequent relapses [age and sex–adjusted HR 1.04 (95% CI, 0.97, 1.11)]. The AUC of the PETVAS in predicting subsequent relapses was 0.60, with a cut point of ≥9 yielding a sensitivity of 47% and a specificity of 74%. Results from previous studies on the value of PET scans for predicting clinical relapse have been inconclusive. While one study failed to demonstrate the predictive value of PET in 35 patients with GCA [31], in the study by Grayson *et al.* [13], the burden of arterial uptake during clinical remission expressed by the PETVAS predicted relapses during the follow-up. However, a threshold score to predict clinical relapse was not proposed. Further studies are needed to assess the role of PET/CT, alone or with other clinical and/or laboratory characteristics, in predicting subsequent clinical relapses.

This study has important limitations to consider. Its retrospective nature is one of the main limitations. However, this was balanced by the fact that our patients were homogeneously followed up at a single centre with a standardized follow-up protocol. Furthermore, because patients with LVV could be enrolled at any time in the disease course, some patients were already diagnosed and treated when they were included in the study. Moreover, the use of GCs by a large number of patients and differences in the disease duration might have interfered with PET/CT imaging results. Also, the use of NIH criteria to evaluate disease activity represents a limitation, because these criteria have not been validated in LV-GCA. Finally, the inclusion of PET/CT findings to identify relapses can lead to an overestimation of its ability to predict disease flares. However, the failure to identify any association between PET/CT performed during remission and subsequent relapses makes this concern irrelevant. The main strengths are the number of consecutive PET/CT scans reviewed (the largest to date), the large number of patients included, with a similar proportion of TAK and LV-GCA patients, which enabled comparative assessment across different forms of vasculitis, and the length of the follow-up.

In conclusion, the visual PET/CT grading scale and the PETVAS had moderate accuracy to distinguish active LVV from remission, with better performance in TAK than in LV-GCA. These findings indicate that the clinical judgement of the rheumatologist expert in vasculitis who integrates symptoms, laboratory markers and morphologic and functional imaging remains a critical step in the assessment of disease activity in LVV. The PETVAS did not predict subsequent disease relapses, and further prospective studies with a validated clinical definition of disease activity are needed to assess the role of PET/CT in predicting disease outcome.

## Supplementary Material

keac125_Supplementary_Data

## Data Availability

Data are available upon reasonable request by corresponding author.
