# Supplementary material for: The role of PET/CT in disease activity assessment in patients with large vessel vasculitis
Source: Rheumatology (Oxford). 2022 Mar 8;61(12):4809–16. doi: 10.1093/rheumatology/keac125 (PMC9707005; doi:10.1093/rheumatology/keac125)
Supplement: keac125_Supplementary_Data [file keac125_supplementary_data.docx]

Supplementary Table S1. Clinical manifestations of patients with active disease and relapses.

|  | **LV-GCA** | **TAK** | **Total** |
| --- | --- | --- | --- |
| **Active disease** |  |  |  |
| PET/CT scans, no. (%) | 71 (57) | 54 (43) | 125 (100) |
| Vascular symptoms, no. (%) | 22 (31) | 30 (56) | 52 (42) |
| Systemic symptoms, no. (%) | 46 (65) | 18 (33) | 64 (51) |
| Cranial symptoms, no. (%) | 16 (23) | 3 (6) | 19 (15) |
| Visual symptoms, no. (%) | 2 (3) | 3 (6) | 5 (4) |
| PMR symptoms, no. (%) | 18 (25) | 0 (0) | 18 (14) |
| **Relapses** |  |  |  |
| Patients, no. (%) | 15 (44) | 19 (56) | 34 (100) |
| Vascular symptoms, no. (%) | 2 (13) | 5 (26) | 7 (21) |
| Systemic symptoms, no. (%) | 6 (40) | 6 (32) | 12 (35) |
| Cranial symptoms, no. (%) | 1 (7) | 0 (0) | 1 (3) |
| Visual symptoms, no. (%) | 0 (0) | 0 (0) | 0 (0) |
| PMR symptoms, no. (%) | 4 (27) | 1 (5) | 5 (15) |
| Radiographic progression, no. (%) | 10 (67) | 16 (84) | 26 (76) |

The PETVAS was not associated with subsequent relapses in TAK patients: age and sex adjusted HR 1.07 (95% CI 0.97, 1.17), p=0.161. The AUC of PETVAS in predicting subsequent relapses was 0.59 (95% CI 0.45, 0.73), Figure S1A. A PETVAS ≥9 showed 52.6% sensitivity and 65.8% specificity in predicting subsequent relapses.

Supplementary Figure S1A. AUC of PETVAS in predicting subsequent relapses in TAK

The PETVAS was not associated with subsequent relapses in GCA patients: age and sex adjusted HR 1.02 (95% CI 0.93, 1.13), p=0.634. The AUC of PETVAS in predicting subsequent relapses was 0.60 (95% CI 0.47, 0.73), Figure S1B. A PETVAS ≥9 showed 40% sensitivity and 81.8% specificity in predicting subsequent relapses.

Supplementary Figure S1B. AUC of PETVAS in predicting subsequent relapses in GCA
